# Circulating CXCR3^+^ Tfh cells positively correlate with neutralizing antibody responses in HCV-infected patients

**DOI:** 10.1038/s41598-019-46533-w

**Published:** 2019-07-12

**Authors:** Jian Zhang, Wenpei Liu, Bo Wen, Ting Xie, Ping Tang, Yabin Hu, Liyan Huang, Kun Jin, Ping Zhang, Ziyan Liu, Ling Niu, Xiaowang Qu

**Affiliations:** 1 Translational Medicine Institute, The First People’s Hospital of Chenzhou, University of South China, Chenzhou, Hunan 423000 China; 2 Affiliated The First People’s Hospital of Chenzhou, Southern Medical University, Chenzhou, Hunan 423000 China

**Keywords:** Viral hepatitis, Viral infection

## Abstract

Circulating T follicular helper (cTfh) cells have been identified as counterparts of germinal center Tfh (GC Tfh) cells in humans and can support T-dependent B cell maturation and antibody production *in vitro*. However, the role of cTfh cells in neutralizing antibody (nAb) responses in HCV infection remains unclear. Here, we characterized the phenotype and function of cTfh cells and demonstrated the associations of cTfh cells and their subsets with nAb responses in HCV infection. A total of 38 HCV-infected individuals and 28 healthy controls were enrolled from a pool of injection drug users. The frequency and function of blood Tfh cells were analyzed by flow cytometry. The titers and breadths of serum nAbs were measured using HCV pseudo-particle neutralization assays. Herein, we report several key observations. First, HCV infection skewed cTfh toward CXCR3^+^ cTfh cell differentiation. Second, the frequency of CXCR3^+^ cTfh cells positively correlated with HCV nAb titers and breadths. Third, CXCR3^+^ cTfh cells showed higher expression of Tfh-associated molecules (PD-1, ICOS, IL-21, Bcl-6) compared with CXCR3^−^ cTfh cells from individuals with HCV infection. Coculture of cTfh cells and autologous memory B cells *in vitro* indicated that CXCR3^+^ cTfh cells show a superior ability to support HCV E2-specific B cell expansion compared with CXCR3^−^ cTfh cells from individuals with HCV infection. HCV infection skews cTfh cells toward CXCR3-biased Tfh cell differentiation, which positively correlates with the magnitude and breadth of the HCV nAb response. It is our hope that these findings will provide insights for the rational design of a nAb-based HCV vaccine.

## Introduction

Neutralizing antibodies (nAbs) play an important role during HCV infection, as a robust early induction of appropriate nAb levels in acute infection contributes to spontaneous HCV clearance and to the prevention of reinfection^1–3^. Broad nAbs are also associated with improved clinical parameters in chronic HCV infection, and higher titers of nAbs promote the natural resolution of chronic HCV infection^4–6^.

T follicular helper (Tfh) cells are a CD4^+^ T cell subset specialized to regulate the types of antibody production that occur in the germinal center (GC)^7,8^. These cells, which reside in the GC, exhibit a CXCR5^+^ PD-1^hi^ ICOS^hi^ surface phenotype and express high levels of the master transcription factor Bcl-6 to regulate the differentiation of antigen-specific memory B cells and plasma cells by secreting the cytokine IL-21^9–13^. Circulating Tfh (cTfh) cells resemble GC Tfh cells because they also expresses low levels of PD-1, ICOS and Bcl6, and these cells exhibit a memory phenotype and serve as a counterpart to GC Tfh cells^14,15^. Based on CXCR3 and CCR6 expression, cTfh cells can be divided into three major subsets: Th1-like cTfh (Tfh1: CXCR3^+^ CCR6^−^) cells, Th2-like cTfh (Tfh2: CXCR3^−^ CCR6^−^) cells and Th17-like cTfh (Tfh17: CXCR3^−^ CCR6^+^) cells^15,16^. According to PD-1, ICOS and CCR7 expression, these subsets can be further divided into many functional populations^15,16^. Recently, accumulating evidence has shown that human blood CXCR3^−^ cTfh (including Tfh2 and Tfh17) cells are major functional counterparts of GC Tfh cells that efficiently induce naïve B cells to produce antibodies *in vitro*, whereas CXCR3^+^ cTfh cells lack these properties^14–16^. Consistent with these findings, Locci M. *et al*. reported that circulating PD-1^+^ CXCR3^−^ CXCR5^+^ memory Tfh cells are highly functional and correlate with a broad neutralizing HIV antibody response in HIV^+^ donors^17^. In contrast, Martin-Gayo E. *et al*. reported that circulating PD-1^lo^ CXCR3^+^ CXCR5^+^ Tfh-like cells in HIV controllers correlate with nAb breadth^18^. Another study showed that the frequencies of circulating Th1-based helper cells in acute HIV infection correlate with the development of HIV-specific antibody responses^19^. The administration of trivalent split-virus influenza vaccines was shown to induce the temporary accumulation of circulating ICOS^+^ CXCR3^+^ CXCR5^+^ CD4^+^ T cells, and up to 60% of these cells were specific for influenza antigens and correlated with an increase in preexisting antibody titers. Further studies showed that these cells contributed to the generation of high-avidity antibodies following influenza vaccination^20,21^. Iyer SS. *et al*. also reported that a gp140 boost induced by an MVA vector-encoded SIV vaccine skewed Tfh cells toward CXCR3 differentiation in the GC, which was strongly associated with the longevity, avidity and neutralization potential of the vaccine-elicited antibody response^22^. It is clear that cTfh cells play a critical role in antibody responses; however, the functionality of these circulating cell subsets may be context dependent and may rely on the virus type, infection state or the antigen used for immunization.

HCV infection usually induces T-cell-dependent antibody responses. Raziorrouh B. *et al*. recently reported that HCV NS4-specific CD4^+^ T cells from patients with acute HCV infection express markers of Tfh cells and secrete interleukin-21 in response to HCV exposure; moreover, these cells highly express the chemokine receptor CXCR3, which positively correlates with anti-HCV NS4 antibodies^23^. Chronic HCV infection partially impairs IL-21 secretion from cTfh cells, but these cells are still capable of supporting memory B cell differentiation and antibody production *in vitro*^24^. However, the impact of HCV infection on cTfh cell differentiation and the role of cTfh cells or their subsets in nAb responses are not yet clear. Here, we show that HCV infection skewed cTfh cells toward CXCR3-biased Tfh cell differentiation, which positively correlated with the magnitude and breadth of the HCV nAb response. It is our hope that these findings will provide clues for the rational design of an HCV vaccine.

## Materials and Methods

### Participants and sampling

In total, 38 HCV-infected individuals and 28 healthy controls were enrolled in this study during 2015–2017 in Chenzhou, Hunan Province; all subjects were identified from a pool of injection drug users (IDUs). Each participant was interviewed using a structured questionnaire to collect demographic data and environmental exposure history. This study was approved by the Ethics Committee of The First People’s Hospital of Chenzhou (No. 2015002) and followed the principles established by the Declaration of Helsinki. All participants enrolled in the study provided written informed consent.

After blood samples were collected, serum and peripheral blood mononuclear cells (PBMCs) were immediately isolated and stored at −80 °C or in liquid nitrogen. All subjects were naïve to antiviral treatment, and individuals with ongoing HBV, HDV, or HIV infection were excluded. The clinical parameters are shown in Table 1; the HCV-infected individuals and healthy controls were age- and sex-matched and showed no difference in ALT/AST levels. According to the questionnaire, the average infection time was estimated at 18.4 years (range, 12.3 to 29.6 years). Among these HCV-infected individuals, 30 individuals were anti-HCV IgG positive and HCV RNA positive (Chronic), the remaining 8 individuals were anti-HCV IgG positive and HCV RNA negative (Recovery). The average of HCV virus titer was 4.19 × 10^5^ copies/ml, and the major HCV subtypes were 6a (36.8%), 3a (18.4%) and 3b (15.8%). Anti-HCV IgG negative and HCV RNA negative and age- and sex-matched healthy controls were included. For all of these subjects, anti-HIV IgG, HBsAg-positive individuals were excluded from this study.

**Table 1:** Clinical information of HCV-infected individuals and healthy controls.

	Healthy controls (n = 28)	HCV-infected individuals (n = 38)	*P* Value^Δ^
Age (years)	36.06 (29.25~46.16)	39.30 (35.02~43.71)	0.166
Sex (Male/Female)	28/0	35/3	0.256
ALT (U/L)	26.60 (20.60~37.95)	40.50 (22.30~70.00)	0.133
AST (U/L)	25.00 (21.70~32.20)	33.00 (22.70~42.30)	0.186
HCV RNA (×10^5^ copies/mL)	−	4.19 (0.60~7.70)	
Anti-HCV IgG	−	+	
HCV genotype (1a/1b/3(a+b)/6a/N.D)(number)	−	1/2/7/6/14/8	

^Δ^Mann-Whitney U test was used for age and ALT/AST comparison, and Fisher’s exact test was used for sex distribution between groups. The data are presented as the median and range. N.D, not determined.

### Neutralization assay

HCV pseudoparticles (HCVpps) were generated by cotransfecting HEK-293 cells with one of 6 subtypes of HCV E1E2 protein-encoding plasmids (Genotype 1a: strain H77c, accession number AF011751.1; Genotype 1b: strain HC-J4, accession number AF054255.1; Genotype 2a: strain J6, accession number JQ745650.1; Genotype 3a: strain S52, accession number GU814263.1; Genotype 4a: strain ED43, accession number GU814266.1; Genotype 5a: strain SA13, accession number AF064490.1) and a lentiviral vector containing a luciferase reporter. Culture supernatants were collected after 3 days. nAb responses, including antibody titers and cross-activity, were determined as previously described^4^. In brief, HCVpps were mixed with diluted serum (1:100, 1:400, 1:1600, 1:6400 diluted with complete DMEM), after which the mixed HCVpp and serum dilutions were incubated for 1 hour at 37 °C in an incubator containing 5% CO_2_ and then used to infect Huh7.5 cells. At 4 hours after inoculation, the culture supernatants were removed, and fresh medium was added to continue culturing. At 3 days after HCVpp infection, the luciferase activity of lysed HCVpp-infected Huh7.5 cells was measured using a firefly luciferase assay system (Promega, Madison, WI, USA) to assess the reduction in infectivity.

### Flow cytometry

Blood samples from the participants were collected in tubes containing a sodium citrate anticoagulant. PBMCs were immediately isolated via Ficoll density gradient centrifugation (GE Healthcare Bio-Sciences AB, Kontaktuppgifter, Sweden) according to the manufacturer’s protocol and stored in liquid nitrogen using a programmed cooling method. For flow cytometry analysis, cryopreserved PBMCs were placed at 37 °C immediately after removal from liquid nitrogen storage to promote recovery. All samples were plated in complete RPMI 1640 medium supplemented with 10% FBS, 1% pen/strep, and L-glutamine, followed by incubation overnight at 37 °C in an incubator containing 5% CO_2_ before staining. For cell surface staining, 1 × 10^6^/mL PBMCs were first labeled with a LIVE/DEAD^®^ Fixable Blue Dead Cell Stain Kit (Thermo Fisher Scientific, Waltham, MA, USA) to exclude dead cells and then treated with Fc Block (BioLegend, San Diego, CA, USA) to block nonspecific binding prior to staining with fluorescently labeled antibodies, followed by staining with a titrated amount of antibodies in 96-well V-bottom plates at 4 °C for 30 min. To characterize the expression of transcription factors in Tfh cells, a Foxp3 Transcription Factor Staining Buffer Kit (eBioscience, San Diego, CA, USA) was used for cell permeabilization and antibody staining against transcription factors.

The antibodies used in this study were as follows: FITC mouse anti-human CD19 (HIB19), FITC mouse anti-human CD45RA (HI100), FITC mouse anti-human IL-17a (N49-653), PE mouse anti-human CXCR3 (1C6), PE-Cy7 mouse anti-human IFN-γ (B27), BUV 737 mouse anti-human CD4 (SK3), BUV 737 mouse anti-human CD8 (SK1), and APC-Cy7 mouse anti-human HLA-DR (G46-6) from BD Biosciences (Franklin Lake, NJ, USA); FITC mouse anti-human PD-1 (EH12.2H7), PerCP-Cy5.5 mouse anti-human CD45RA (HI100), PerCP-Cy5.5 mouse anti-human IL-10 (JES3-9D7), APC mouse anti-human CD138 (MI15), APC mouse anti-human IL-21 (3A3-N2), and APC-Cy7 mouse anti-human CD3 (SK7) from BioLegend (San Diego, CA, USA); and PE-eFluor 610 mouse anti-human CXCR5 (MU5UBEE), APC mouse anti-human CXCR5 (MU5UBEE), PE-Cy7 mouse anti-human T-bet (4B10), APC mouse anti-human Bcl-6 (BCL-UP), APC mouse anti-human ICOS (ISA-3), APC mouse anti-human Ki67 (20Raj1), and APC-eFluor 780 mouse anti-human CD38 (HIT2) from Thermo Fisher Scientific (Waltham, MA, USA). Cell population gating was performed based on the mean fluorescence intensity “minus one” (FMO) and unstained controls. Samples were acquired in a MoFlo XDP flow cytometer (Beckman Coulter, Brea, CA, USA) immediately after staining. All subsequent data analyses were performed with FlowJo 10.0 software (Tree Star, San Carlos, CA, USA).

### Intracellular cytokine staining

To determine the cytokine secretion capacity of Tfh cells, PBMCs or sorted Tfh cells were seeded in 96-well U-bottom plates (Thermo Fisher Scientific, Waltham, MA, USA) and stimulated with 50 ng/mL PMA (Sigma-Aldrich, St. Louis, MO, USA) and 1 μM/mL ionomycin (Sigma-Aldrich, St. Louis, MO, USA) for 5 hours. GolgiStop (BD Biosciences, Franklin Lake, NJ, USA) was added 1 hour after stimulation. For flow cytometry assessment, cells were stained with surface antibodies and fixed using a Cytofix/Cytoperm fixation solution (Beckman Coulter, Brea, CA, USA) for intracellular cytokine staining according to the manufacturer’s recommendations.

### Cell sorting

For Tfh and memory B cell coculture, at least 10 million PBMCs from HCV-infected individuals were sorted. Live CXCR3^+^ Tfh and CXCR3^−^ Tfh cells were sorted from the PBMCs as CXCR3^+^ CXCR5^+^ CD4^+^ T or CXCR3^−^ CXCR5^+^ CD4^+^ T cells, respectively, after staining with CD3 APC-Cy7, CD4 APC, CXCR5 PE-eFluor 610, and CXCR3 PE. Autologous memory B cells were sorted from the PBMCs as CD3^−^ CD19^+^ CD27^+^ B cells after staining with CD27 PE-cy7, CD3 APC-Cy7, and CD19 FITC. Sorting was conducted using a MoFlo XDP sorter (Beckman Coulter, Brea, CA, USA).

### Coculture

To test the functionality of cTfh cells in supporting B cell differentiation *in vitro*, sorted memory B cells (2 × 10^4^) were cocultured with 2 × 10^4^ autologous CXCR3^+^ Tfh cells or 2 × 10^4^ autologous CXCR3^−^ Tfh cells in 96-well U-bottom plates (Thermo Fisher Scientific, Waltham, MA, USA) in RPMI1640 medium supplemented with 10% fetal bovine serum in the presence of endotoxin-reduced staphylococcal enterotoxin B (SEB) (1 µg/ml) (Toxin Technology, Sarasota, FL, USA). On day 7, the levels of IgM, IgG and IgA produced in the culture supernatants were determined with corresponding ELISA kits (Thermo Fisher Scientific, Waltham, MA, USA). Plasma cells (CD3^−^ CD19^+^ CD38^+^ CD138^+^) and HCV E2-specific B cells (CD3^−^ CD19^+^ HCV E2c Probe^+^) were analyzed by flow cytometry after 7 days of coculture, to assess the helper function of CXCR3^+^ and CXCR3^−^ Tfh cells with autologous memory B cells.

### Measurement of HCV E2-specific B cells

To measure HCV E2 antigen-specific B cells, the E2c glycoprotein (amino acids 412 to 645, genotype 1b) was expressed in HEK293 mammalian cells by transfecting plasmids encoding E2c (kindly provided by Mansun Law, Scripps Research Institute, CA, USA). The HCV E2c tetramer was generated as follows. Biotinylated E2c monomers were incubated with allophycocyanin-labeled streptavidin (Molecular Probes; Thermo Fisher Scientific, Rochester, NY) at a molar ratio of 4:1. Fluorescently labeled streptavidin reagent was added to the E2c monomer in five aliquots, each followed by 20 min of incubation at 4 °C with shaking in the dark. The frequency of HCV E2c-specific B cells was measured using the HCV E2c tetramer as a probe, as previously reported^25^. To avoid false-positive staining due to potential binding of the HCV E2 antigen with CD81 molecules expressed on B cells, cells were treated with an anti-CD81 blocking antibody (JS-81) before HCV E2c tetramer staining. To define the background and baseline of E2c tetramer staining, we compared the HCV E2c-specific B cell level in PBMCs of healthy controls and HCV-infected individuals by staining with HCV E2c probe *ex vivo*. As shown in Supplementary Fig. 1, the HCV E2c tetramer assay displayed high sensitivity and specificity, which is consistent with previous data in chronic HCV infection reported by Maude Boisvert using the E2 tetramer assay^25^.

### Statistical analysis

All results are presented as the median and interquartile range. The Mann-Whitney U test was used for comparisons of two independent samples, and the paired t-test was used to analyze two matched samples. Fisher’s exact test was employed to compare the distribution among the groups. Spearman’s rank correlation coefficient was used to evaluate the relationship between two variables. Significance was set at *P* < 0.05. All statistical calculations were performed with either the SPSS 19.0 program (Chicago, IL, USA) or Prism 7 (GraphPad Software, Inc., USA).

## Results

### HCV infection skews cTfh cells toward CXCR3^+^ cTfh cell differentiation

**Figure 1:**
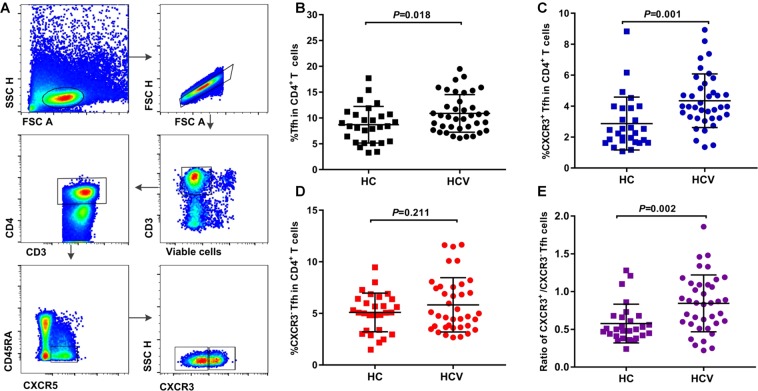
HCV infection skewed cTfh cells toward CXCR3^+^ cTfh differentiation. (**A**) Representative flow cytometry plots of the gating strategy of CXCR3^+^ cTfh and CXCR3^−^ cTfh cells. (**B–E**) Comparison of the percentages of cTfh, CXCR3^+^ cTfh, and CXCR3^−^ cTfh cells as well as the ratio of CXCR3^+^/CXCR3^−^ cTfh cells among CD4^+^ T cells between healthy controls (HC, n = 28) and HCV-infected individuals (HCV, n = 38). The data are shown as the median and interquartile range. The Mann-Whitney U test was used for comparisons between two groups.

To demonstrate the impact of HCV infection on cTfh cell differentiation, blood Tfh cells and their subsets from 38 HCV-infected individuals and 28 age- and sex-matched healthy controls from a pool of IDUs were analyzed based on CXCR5 expression on CD4^+^ T cells (Fig. 1A). Compared with healthy controls, HCV-infected individuals showed a significant increase in the frequency of cTfh cells (*P* = 0.018) (Fig. 1B). Furthermore, HCV infection mainly increased the frequency of CXCR3^+^ cTfh cells (*P* = 0.001), but not that of CXCR3^−^ cTfh cells (*P* = 0.211), compared with healthy controls (Fig. 1C,D). The ratio of CXCR3^+^/CXCR3^−^ cTfh cells in HCV-infected individuals was also elevated compared with healthy controls (*P* = 0.002) (Fig. 1E). These results suggested that HCV infection promoted cTfh cell expansion and skewed cTfh cells toward CXCR3^+^ cTfh cell differentiation, which is consistent with a report showing that most antigen-specific CD4^+^ T cells express CXCR3 and exhibit similar properties to Tfh cells in acute HCV infection, based on MHC II tetramer staining^23^.

### CXCR3^+^ cTfh cells positively correlate with the magnitude and breadth of the HCV neutralizing antibody response

**Figure 2:**
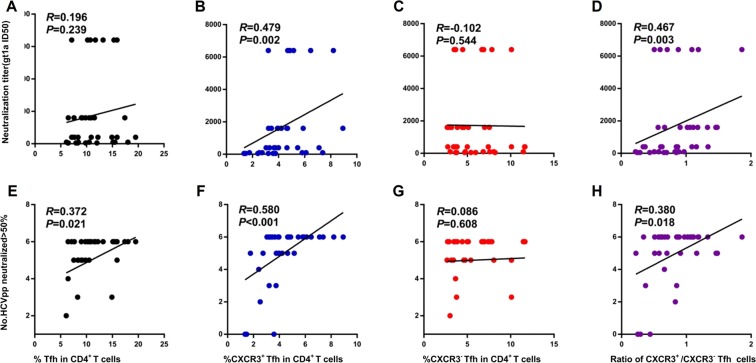
Relationship of cTfh cells and their subsets with HCV neutralizing antibody responses. (**A–D**) Correlations of the neutralization titer (genotype 1a is shown as a representative) with cTfh cells (**A**), CXCR3^+^ cTfh cells (**B**), CXCR3^−^ cTfh cells (**C**), and the ratio of CXCR3^+^/CXCR3^−^ Tfh cells (**D**) in CD4^+^ T cells in HCV infection (n = 38). (**E-F**) Correlations of the neutralization breadth with cTfh cells (**E**), CXCR3^+^ cTfh cells (**F**), CXCR3^−^ cTfh cells (**G**), and the ratio of CXCR3^+^/CXCR3^−^ Tfh cells (**H**) in CD4^+^ T cells in HCV infection (n = 38). Neutralization titers were calculated by combining fourfold dilutions of serum (dilution started from 1:100) with HCVpps. The neutralizing breadth was indicated by the number of HCVpps neutralized >50%; positive neutralization was categorized as a reduction in infectivity by 50% in this HCVpp system. Spearman *R* and *P* values are indicated.

As shown above, HCV infection drove cTfh cells toward CXCR3^+^ cTfh cell differentiation. To assess the relationship of CXCR3^+^ cTfh cells with HCV nAb responses, we first screened serum nAb responses. nAb titers and breadth were evaluated according to their titers and cross-activity against different HCV genotype pseudoparticles (Supplementary Table 1) as described previously^4^. Association analysis of the frequencies of cTfh cells and their subsets with nAb titers showed that CXCR3^+^ cTfh cells positively correlated with the HCV nAb titer (*R* = 0.479, *P* = 0.002, HCVpp gt 1a as a representative) (Fig. 2B). CXCR3^+^ cTfh cells also showed positive correlations with the neutralizing titers of all HCV subtypes (Supplementary Table 2). There were no associations of cTfh cells or CXCR3^−^ cTfh cells with nAb titers in HCV infection (Fig. 2A,C, Supplementary Table 2). Consistent with the results shown in Fig. 1, an increased ratio of CXCR3^+^/CXCR3^−^ cTfh cells also showed a positive correlation with nAb titers (*R* = 0.467, *P* = 0.003) (Fig. 2D, Supplementary Table 2). Cross-activities represent the nAb breadth; in this study, we tested the potential neutralizing activities of each serum sample from a patient with HCV infection against 6 HCV subtypes (gt 1a, 1b, 2a, 3a, 4a and 5a), and the neutralizing breadth is indicated in Supplementary Table 1. The frequencies of both cTfh cells and CXCR3^+^ cTfh cells showed positive correlations with nAb breadth (*R* = 0.372, *P* = 0.021; *R* = 0.580, *P* < 0.001, respectively) (Fig. 2E,F). There was no correlation between the frequency of CXCR3^−^ cTfh cells and antibody breadth (Fig. 2G). The ratio of CXCR3^+^/CXCR3^−^ cTfh cells also showed a positive correlation with nAb breadth (*R* = 0.380, *P* = 0.018) (Fig. 2H). These individuals included 8 HCV-recovered patients; to more accurately address the relationship of cTfh cells with nAb response in chronic HCV infection, we also analyzed the correlations of cTfh cells and subsets with nAb titer and breadth in chronic HCV infection. As expected, similar results were found (Supplementary Fig. 2).

Whether CXCR3-biased cTfh cells contribute to the antibody response remains controversial. Most studies have indicated that Th1-like cTfh cells lack the helper function with B cells. However, recent studies have shown that CXCR3-biased cTfh cells or populations (based on PD-1 or ICOS expression) actually correlate with antibody responses and show helper function under viral infection or vaccination^14,15,17,18,20^. To further test the relationship of cTfh cell populations with antibody responses, based on PD-1 and CXCR3 expression, we analyzed the correlations of PD-1^+^ CXCR3^+^, PD-1^−^ CXCR3^+^, PD-1^+^ CXCR3^−^ and PD-1^−^ CXCR3^−^ cTfh cell populations with antibody responses. We found that PD1^−^ CXCR3^+^ cTfh cells correlated not only with HCV nAb strength but also with HCV nAb breadth; however, PD1^+^ CXCR3^+^ cTfh correlated only with HCV nAb breadth but not with antibody strength (Supplementary Table 3).

### CXCR3^+^ cTfh cells show distinct immunophenotypic properties compared with CXCR3^−^ cTfh cells in HCV infection

**Figure 3:**
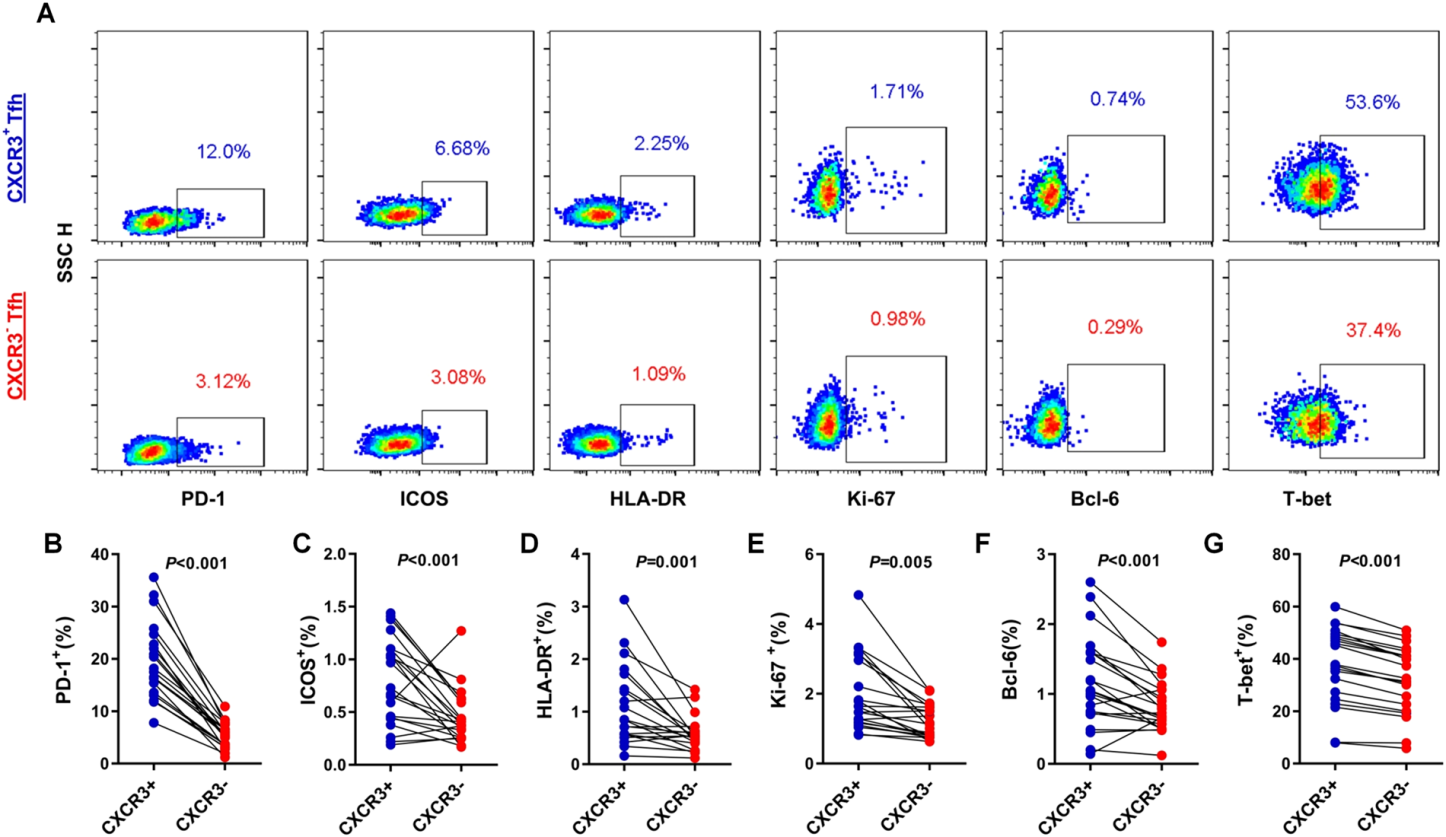
Comparison of the phenotypes of CXCR3^+^ cTfh and CXCR3^−^ cTfh cells from individuals with HCV infection. (**A**) Representative flow cytometry plots of the phenotypes of CXCR3^+^ cTfh and CXCR3^−^ cTfh cells (n = 20). (**B,C**) Expression of PD-1 and ICOS in CXCR3^+^ cTfh and CXCR3^−^ cTfh cells (n = 20). (**D,E**) Expression of HLA-DR and Ki-67 in CXCR3^+^ cTfh and CXCR3^−^ cTfh cells (n = 20). (**F,G**) Expression of the transcription factors Bcl-6 and T-bet in CXCR3^+^ cTfh and CXCR3^−^ cTfh cells (n = 20). The paired t-test was used for the analysis.

To determine why CXCR3^+^ cTfh cells, but not CXCR3^−^ cTfh cells, correlate with HCV nAb responses in HCV infection, we compared the expression levels of Tfh cell linage-associated molecules (PD-1, ICOS), activation and proliferation markers (HLA-DR, Ki-67) and transcription factors (Bcl-6, T-bet) between CXCR3^+^ cTfh cells and CXCR3^−^ cTfh cells from 20 individuals with HCV infection (Fig. 3A). CXCR3^+^ cTfh cells showed significantly higher PD-1 and ICOS expression than matched CXCR3^−^ cTfh cells (*P* < 0.001 and *P* < 0.001, respectively) (Fig. 3B,C). CXCR3^+^ cTfh cells also exhibited greater activation and proliferation potential than CXCR3^−^ cTfh cells (*P* = 0.001 and *P* = 0.005, respectively) (Fig. 3D,E). Staining of the transcription factors Bcl-6 and T-bet showed higher expression in CXCR3^+^ cTfh cells compared with CXCR3^−^ cTfh cells (*P* < 0.001 and *P* < 0.001, respectively) (Fig. 3F,G). These results indicate that CXCR3^+^ cTfh cells phenotypically exhibit a better potential to support B cell differentiation than CXCR3^−^ cTfh cells in HCV infection, which may more efficiently contribute to nAb responses.

### CXCR3^+^ cTfh cells show a greater capacity for Tfh-associated cytokine secretion than CXCR3^−^ cTfh cells from individuals with HCV infection

**Figure 4:**
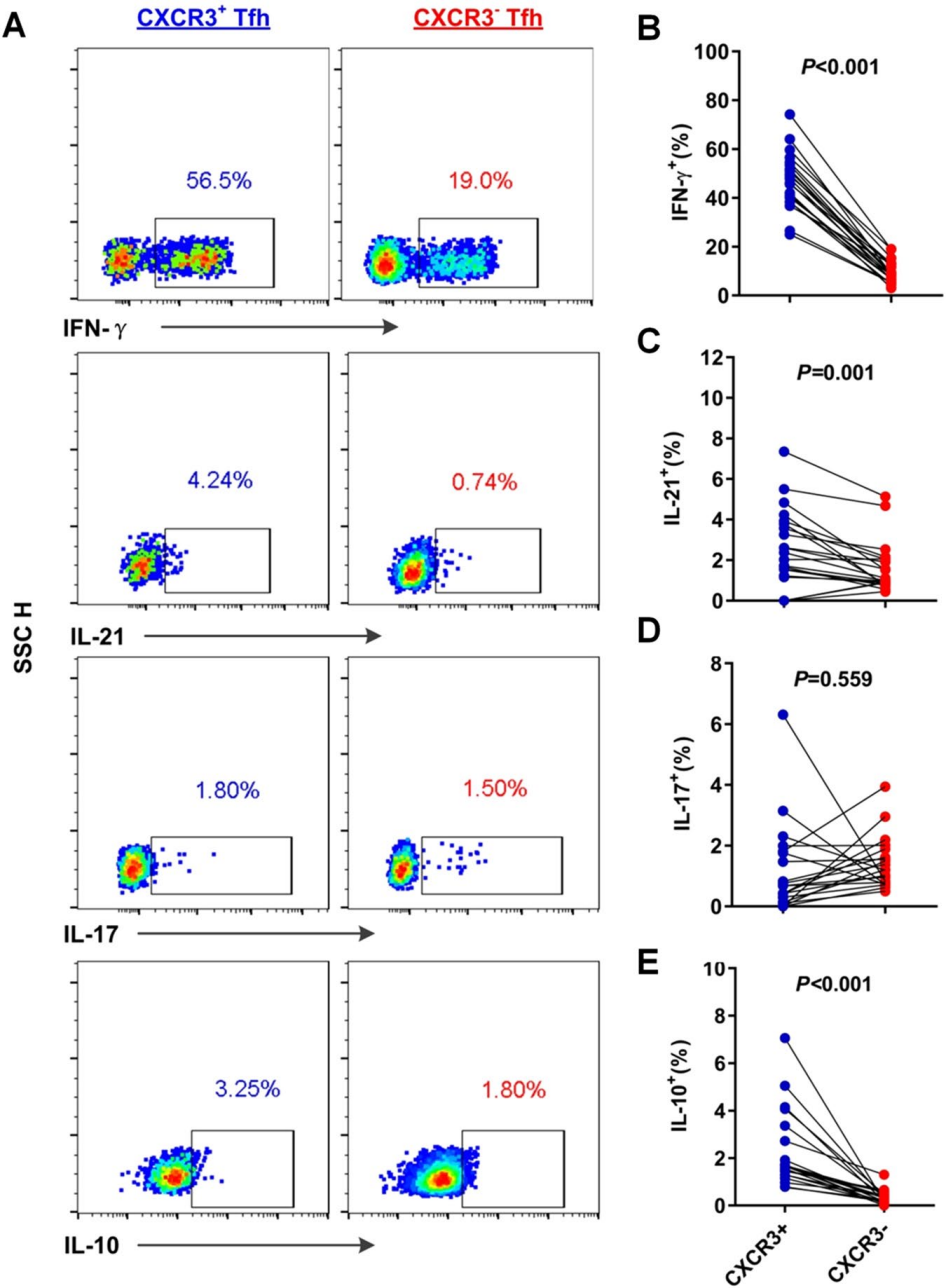
Comparison of cytokine secretion of CXCR3^+^ cTfh and CXCR3^−^ cTfh cells from individuals with HCV infection. (**A**) Representative flow cytometry plots of cytokine expression in CXCR3^+^ cTfh and CXCR3^−^ cTfh cells after stimulation by PMA and ionomycin. Because CD4 expression on T cells was significantly decreased after PMA and ionomycin costimulation, we gated CD8^−^ T cells and regarded them as CD4^+^ T cells for further analysis of cytokine on cTfh cells, (**B–E**) Comparison of the expression levels of IFN-γ (**B**), IL-21 (**C**), IL-17 (**D**), and IL-10 (**E**) between CXCR3^+^ cTfh and CXCR3^−^ cTfh cells from individuals with HCV infection (n = 21). The paired t-test was used for the analysis.

CXCR3^+^ cTfh cells show higher expression of Tfh phenotype-associated molecules than CXCR3^−^ Tfh cells in the context of HCV infection. To further assess the differences in the functionality of CXCR3^+^ cTfh cells and CXCR3^−^ cTfh cells from 21 individuals with HCV infection, Tfh cell-associated cytokine secretion was analyzed in response to PMA and ionomycin stimulation (Fig. 4A). Compared with CXCR3^−^ cTfh cells, CXCR3^+^ cTfh cells expressed significantly higher levels of IFN-γ (*P* < 0.001), IL-21 (*P* = 0.001) and IL-10 (*P* < 0.001) (Fig. 4B–C, E). These cytokines secreted by Tfh cells are required for the maintenance of Tfh cells or plasma cell differentiation^26,27^. Higher cytokine secretion showed that CXCR3^+^ cTfh cells present greater potential functionality than CXCR3^−^ cTfh cells to support B cell differentiation in HCV infection.

### CXCR3^+^ cTfh cells show a greater supporting capacity for antigen-specific B cell expansion than CXCR3^−^ cTfh cells *in vitro*

**Figure 5:**
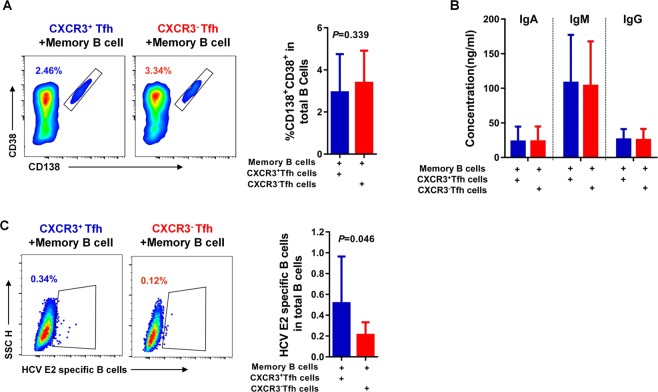
CXCR3^+^ cTfh cells show a superior ability to support HCV-specific B cell expansion compared with CXCR3^−^ cTfh cells from individuals with HCV infection *in vitro*. (**A**) Representative flow cytometry plots and the percentage of plasma cells (CD38^+^ CD138^+^-gated CD3^−^ T cells) after CXCR3^+^ cTfh or CXCR3^−^ cTfh cells were cocultured with autologous memory B cells for 7 days in the presence of SEB (1 µg/ml). After 7 days, B cells were defined as CD3^−^ CD4^−^ cells because plasmablasts/plasma cells can downregulate CD19 and are CD20 negative. (**B**) Comparison of IgA, IgM and IgG production in the supernatant after CXCR3^+^ cTfh cells or CXCR3^−^ cTfh cells were cocultured with autologous memory B cells (n = 7). (**C**) Representative flow cytometry plots of HCV E2-specific B cells and comparison of HCV E2-specific B cells after CXCR3^+^ cTfh cells or CXCR3^−^ cTfh cells were cocultured with autologous memory B cells (n = 6). The paired t-test was used for the analysis.

Several studies have shown that CXCR3-biased cTfh cells promote only the differentiation of memory B cells, but not naïve B cells, into plasma cells *in vitro*^20,21^. To confirm the role of CXCR3^+^ cTfh cells in the HCV nAb response, blood memory B cells and cTfh cells from individuals with HCV infection were cocultured *in vitro*. CXCR3^+^ cTfh cells, CXCR3^−^ cTfh cells and autologous memory B cells were then sorted by flow cytometry. Coculture of either 2 × 10^4^ CXCR3^+^ cTfh cells or 2 × 10^4^ CXCR3^−^ cTfh cells with 2 × 10^4^ autologous memory B cells was conducted in the presence of 1 µg/mL SEB for 7 days. After co-culture, the levels of supernatant antibodies (IgA, IgM and IgG) and the frequencies of plasma cells (CD38^+^ CD138^+^ B cells) and HCV E2-specific B cells were determined by flow cytometry. Our data showed that both CXCR3^+^ cTfh cells and CXCR3^−^ cTfh cells could efficiently support autologous memory B cell differentiation into plasma cells *in vitro*. There was no difference between CXCR3^+^ cTfh cells and CXCR3^−^ cTfh cells with regard to promoting memory B cell differentiation into plasma cells (*P* = 0.339) (Fig. 5A), and the levels of IgA, IgM and IgG antibodies in the supernatants after coculturing CXCR3^+^ cTfh cells or CXCR3^−^ cTfh cells with autologous memory B cells were almost the same (Fig. 5B). To determine the differences between CXCR3^+^ cTfh cells and CXCR3^−^ cTfh cells in the promotion of antigen-specific B cell expansion, we measured the frequencies of HCV E2-specific B cell expansion after coculture. The CXCR3^+^ cTfh cells cocultured with autologous memory B cells showed a higher frequency of HCV E2 antigen-specific B cells than CXCR3^−^ cTfh cells (*P* = 0.046) (Fig. 5C). These data suggest that CXCR3^+^ cTfh cells from HCV infection exhibit a greater ability to promote HCV antigen-specific B cell expansion than CXCR3^−^ cTfh cells. Coculture of cTfh with autologous memory B cells suggested that CXCR3^+^ cTfh cells play an important role in supporting antibody responses in HCV infection.

## Discussion

Here, we report that HCV infection skewed cTfh cells toward CXCR3^+^ cTfh cell differentiation, which positively correlated with the magnitude and breadth of the HCV nAb response in HCV infection. These findings suggest that CXCR3^+^ cTfh cells play a critical role in HCV nAb responses.

HCV infection is usually accompanied by nAb production, which is delayed in subjects that developed chronic infection^3,28^. The production of HCV nAbs is one of the most important immune responses for preventing HCV infection. The early release of nAbs usually correlates with the spontaneous clearance of HCV infection, and cumulative and delayed nAb responses in chronic HCV infection may help to control viral replication and reduce the risk of fibrosis^3,5,29–31^. Human blood Tfh cells have been identified as counterparts of GC Tfh cells and exhibit B cell helper function, and some subsets of cTfh cells correlate with viral nAb responses in viral infection or vaccination^14,17,18,32^. Bijan Raziorrouh identified HCV antigen-specific CD4^+^ T cells with similar properties to Tfh cells associated with anti-HCV production in acute infection^23^. Although chronic HCV infection impairs circulating Tfh cytokine production to some extent, these cytokines still show an impressive function in supporting B cell maturation and antibody production *in vitro*^24^. These studies indicated that cTfh cells could be involved in HCV antibody responses, but there is still no direct evidence showing the fundamental role of cTfh cells in HCV nAb responses.

Here, we showed that HCV infection promotes cTfh expansion, which is observed in multiple types of viral infections in human^23,33,34^. Interestingly, HCV infection skewed cTfh cells toward CXCR3-biased Tfh cell differentiation. During HCV infection, the CXCR3-associated chemokines CXCL9-CXCL11, which recruit CXCR3^+^ T cells and B cells to the inflammatory site in the liver, are highly expressed^35–37^. Regarding chemokine- and cytokine-mediated immune cell trafficking and localization, the CXCL9/10/11 ligand and CXCR3 receptor are required for the optimal generation of interferon-γ-secreting Th1 cells *in vivo*^38,39^. Thus, it is possible that these chemokines promote cTfh differentiation and recruit CXCR3^+^ cTfh cells to the inflammatory site, and the activation and proliferation of these cells occur via interaction with cognate B cells involved in HCV pathogenesis. This possibility is supported by CXCR5-positive staining in liver biopsies from individuals with chronic HCV infection^23^.

CXCR3-biased cTfh cells play an important role in nAb production in HIV-1 controllers and following influenza vaccination^20,21,40^. Here, we report that CXCR3^+^ cTfh cells positively correlated with HCV nAb titers and breadth in HCV infection. In contrast, CXCR3^−^ cTfh cells were found to be unrelated to the nAb response. Interestingly, CXCR3^+^ PD1^−^ cTfh cells not only correlated with HCV nAb strength but also with nAb breadth; however, CXCR3^+^ PD1^+^ cTfh cells only positively correlated with HCV nAb breadth but not with HCV nAb strength. The underling mechanism needs further investigation. Phenotypic and functional analyses have shown that compared with CXCR3^−^ cTfh cells, CXCR3^+^ cTfh cells from individuals with HCV infection exhibit relatively higher expression levels of Tfh-associated molecules, such as PD-1 and ICOS, higher HLA-DR and Ki-67 and superior production of cytokines, such as IL-21, IFN-γ, and IL-10. PD-1, ICOS and Bcl-6 are required to maintain the Tfh cell phenotype and B cell helper function^9,10,15,41–46^. High Ki-67 levels also indicate a greater capacity to help B cells^22^. IL-21 is the key cytokine supporting memory B cell maturation and plasma cell differentiation in the germinal center^12,13,47–49^. Autocrine IFN-γ, IL-10, and IL-17 production by Tfh cells also contributes to B cell maintenance and support functions^50–53^. Coculture of either CXCR3^+^ cTfh or CXCR3^−^ cTfh cells with autologous memory B cells results in equivalent total IgA, IgG and IgM production; however, CXCR3^+^ cTfh cells exhibit a superior ability to promote HCV-specific B cell expansion, albeit at a very low level. These results indicated that CXCR3^+^ cTfh cells present superior potential to support B cell maturation compared with CXCR3^−^ cTfh cells under HCV infection. Tetramer staining showed that most HCV NS4-specific CD4^+^ T cells display similar properties to CXCR3^+^ Tfh cells, which are associated with anti-NS4 antibody responses^23^. Accumulating evidence indicates that HCV infection induces CXCR3 expression on B and T cells, which are enriched in the liver. Both our work and that of Bijan Raziorrouh, *et al*. demonstrate that CXCR3^+^ Tfh cells contribute to HCV-specific antibody responses^23,36,38,54^. In our cohort, all HCV-infected individuals have long-term infection, showing low or undetected HCV RNA and with normal ALT/AST levels. Thus, analysis of different phases of HCV infection will be helpful to fully understand the role of cTfh cells in nAb response. In particular, it would be interesting to examine the role of these CD4 T cells in early infection and determine whether they are associated with the early development of nAbs in those who clear the infection.

In conclusion, our data show that HCV infection skewed cTfh cells toward CXCR3^+^ cTfh differentiation, which positively correlated with the magnitude and breadth of the HCV nAb response. These findings indicate that the induction of CXCR3^+^ cTfh cell differentiation through vaccination may be beneficial for eliciting broad HCV nAb responses.

## Supplementary information

Supplementary information
