# Supplementary material for: Circulating CXCR3+ Tfh cells positively correlate with neutralizing antibody responses in HCV-infected patients
Source: Sci Rep. 2019 Jul 12;9:10090. doi: 10.1038/s41598-019-46533-w (PMC6626020; doi:10.1038/s41598-019-46533-w)
Supplement: Supplementary file 1 — Supplementary information [file 41598_2019_46533_MOESM1_ESM.pdf]

Circulating CXCR3+ Tfh cells positively correlate with neutralizing antibodies responses in HCV infected patients

Jian Zhang[1, #], Wenpei Liu[1,2, #], Bo Wen[1], Ting Xie[1], Ping Tang[1], Yabin Hu[1], Liyan Huang[1], Kun Jin[1], Ping Zhang[1], Ziyan Liu[1], Ling Niu[1], Xiaowang Qu[1,2, *]

[1]Translational Medicine Institute, The First People's Hospital of Chenzhou, University of South China, Chenzhou, Hunan, 423000, China.

[2]Affiliated The First People's Hospital of Chenzhou, Southern Medical University, Chenzhou, Hunan, 423000, China.

Supplementary Table 1. Neutralizing antibody responses (titer and breadth) from individuals with HCV infection

Subject	HCVpp neutralization activity*						HCVpp neutralization breadth
	gt1a	gt1b	gt2a	gt3a	gt4a	gt5a	
B026	6400	400	400	400	400	400	6
A036	100	100	50	6400	6400	6400	5
B040	400	400	400	1600	1600	6400	6
B072	50	50	50	50	50	50	0
B113	1600	1600	1600	400	1600	6400	6
B123	400	1600	400	400	6400	6400	6
A159	50	100	50	100	100	100	4
C202	50	50	50	50	50	50	0
C203	100	100	50	50	50	50	2
C280	50	400	100	100	100	400	5
C384	6400	100	50	6400	6400	1600	5
C387	400	100	400	100	400	1600	6
C423	100	1600	1600	100	100	400	6
D008	50	50	50	50	50	50	0
D110	1600	400	400	400	1600	1600	6

D116	6400	1600	400	400	6400	6400	6
D218	6400	1600	1600	400	1600	1600	6
D316	6400	1600	400	400	1600	1600	6
D342	1600	400	100	100	6400	1600	6
F001	1600	400	100	50	1600	6400	5
F033	100	400	100	100	100	1600	6
F041	100	100	100	100	50	6400	5
F050	1600	400	400	1600	1600	6400	6
F077	1600	50	100	400	400	1600	5
F078	400	100	1600	100	1600	1600	6
F089	1600	1600	400	100	1600	6400	6
F125	1600	400	100	100	1600	6400	6
F210	400	1600	400	1600	1600	6400	6
F281	50	50	400	50	400	1600	3
F317	6400	1600	6400	400	400	6400	6
F385	400	50	400	100	1600	1600	5
MA021	400	1600	400	100	1600	6400	6
MA029	400	1600	1600	1600	400	6400	6
MA050	1600	400	400	400	50	1600	5
MA060	1600	400	1600	400	6400	6400	6
MA102	6400	1600	6400	1600	6400	6400	6
MB036	400	50	50	100	50	400	3
MC027	100	1600	400	400	6400	6400	6

*Data present as HCVpp inhibition endpoint titers and neutralization breadth to the HCVpp of each genotype.

Supplementary Table 2. Correlations of HCV neutralizing antibody titer with cTfh cells or subsets from individuals with HCV infection

	% cTfh in $CD4^+$ T cells	% $CXCR3^+$ cTfh in $CD4^+$ T cells	% $CXCR3^-$ cTfh in $CD4^+$ T cells	Ratio of $CXCR3^+$/$CXCR3^-$ cTfh cells
**gt1a**	（0.196,0.239)	(0.479**,0.002)	(-0.102,0.544)	(0.467**,0.003)
**gt1b**	（0.351*,0.031)	(0.535**,0.001)	(0.135,0.420)	(0.260,0.114)
**gt2a**	（0.306,0.062)	(0.504**,0.001)	(0.042,0.803)	(0.323*,0.048)
**gt3a**	（0.304,0.063)	(0.423**,0.008)	(0.164,0.326)	(0.212,0.201)
**gt4a**	（0.164,0.326)	(0.345*,0.034)	(-0.054,0.749)	(0.371*,0.022)
**gt5a**	（0.199,0.231)	(0.316,0.053)	(-0.010,0.952)	(0.311,0.057)

$^{*}P<0.05$, $^{**}P<0.01$. Data present as *R* and *P* value. Spearman’s correlation was used to evaluate the correlation between different variables

Supplementary Table 3.The relationship of both PD-1 and CXCR3 biased cTfh cells populations with neutralization antibody responses from individuals with HCV infection

	HCVpp neutralization activity						HCVpp neutralization breadth
	gt1a	gt1b	gt2a	gt3a	gt4a	gt5a	
%PD-1$^-$ CXCR3$^+$ Tfh in CD4$^+$ T cells	(0.536**,0.005)	(0.615**,0.001)	(0.665**,0.000)	(0.482*,0.013)	(0.429*,0.029)	(0.502**,0.009)	(0.679**, <0.001)
%PD-1$^+$CXCR3$^+$ Tfh in CD4$^+$ T cells	(0.340,0.089)	(0.298,0.139)	(0.367,0.065)	(0.268,0.186)	(0.271,0.180)	(0.164,0.424)	(0.415*,0.035)
%PD-1$^+$ CXCR3$^-$Tfh in CD4$^+$ T cells	(-0.517,0.007)	(-0.471,0.015)	(-0.376,0.058)	(-0.172,0.400)	(-0.330,0.099)	(-0.338,0.091)	(-0.387,0.051)
%PD-1$^-$ CXCR3$^-$ Tfh in CD4$^+$ T cells	(0.030,0.883)	(0.157,0.444)	(0.199,0.330)	(0.199,0.329)	(0.057,0.782)	(0.225,0.269)	(0.127,0.537)

*P<0.05, **P<0.01. Data present as R and P value. Spearman's correlation was used to evaluate the correlation between different variables

A

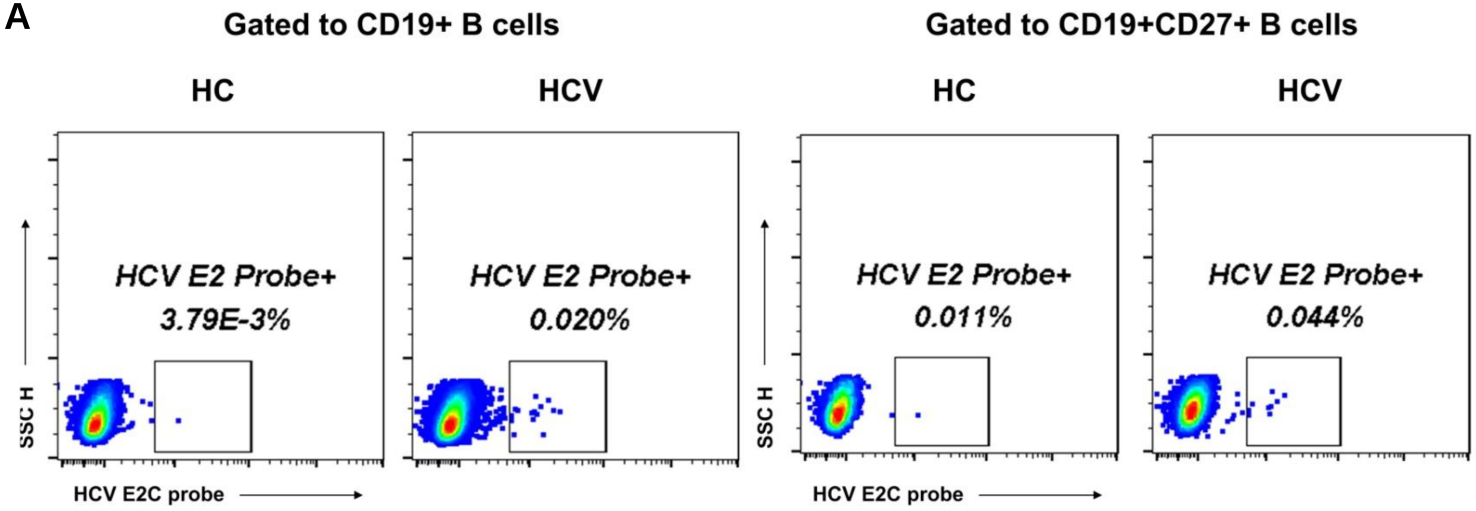

B

	Healthy controls(n=10)	HCV individuals(n=11)	*P*
%HCV E2 specific B cells in $CD19^+$ B cells	0.009(0.006,0.012)	0.032(0.016,0.039)	<0.001
%HCV E2 specific B cells in $CD19^+$ $CD27^+$ B cells	0.02(0,0.025)	0.053(0.029,0.153)	0.003

Supplementary Fig 1. The frequency of HCV E2-specific B cells in HCV patients and healthy controls. (A) Representative flow cytometry plots of HCV E2-specific B cells staining in HCV infected patients and healthy controls. (B) Comparison of the frequency of HCV E2-specific B cells in total B cells ($CD19^+$) and in memory B cells ($CD19^+$ $CD27^+$) between HCV infected patients and healthy controls. Data present as median and interquartile range. Mann-Whitney U test was used for the analysis. $P<0.05$ means significantly different between groups.

.

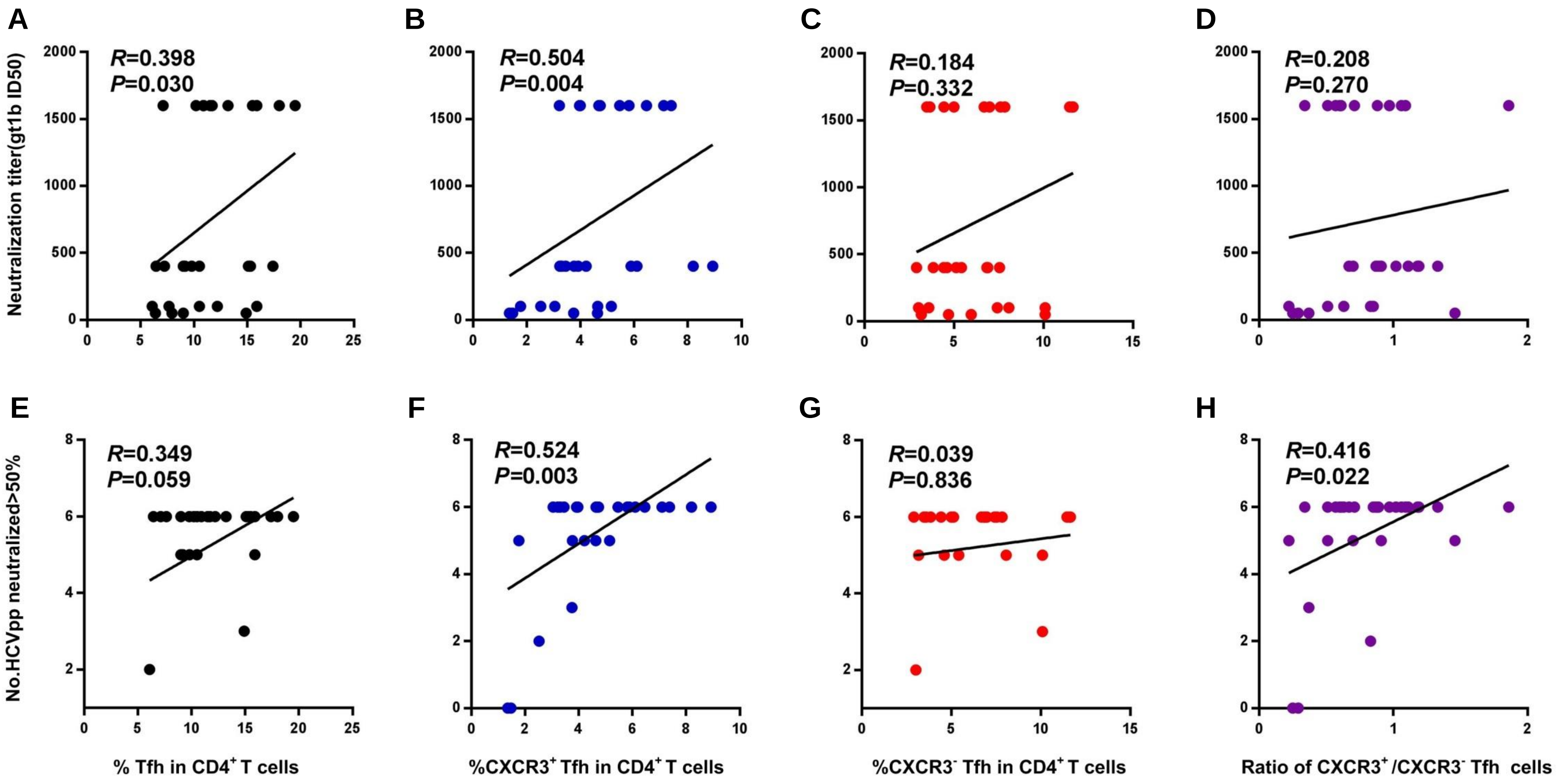

Supplementary Fig 2. Relationship of cTfh cells and subsets with HCV neutralizing antibody responses in chronic HCV infected individuals. (A-D) Correlations of the neutralization titer (genotype 1b as represent) with cTfh cells (A), $CXCR3^+$ cTfh cells (B), $CXCR3^-$ cTfh cells (C), and the ratio of $CXCR3^+$ /$CXCR3^-$ Tfh cells (D) in $CD4^+$ T cells in chronic HCV infection (n=30). (E-F) Correlations of the neutralization breadth with cTfh cells (E), $CXCR3^+$ cTfh cells (F), $CXCR3^-$ cTfh cells (G), and the ratio of $CXCR3^+$ /$CXCR3^-$ Tfh cells (H) in $CD4^+$ T cells in chronic HCV infection (n=30). Neutralization titers were calculated by endpoint dilution of serum (dilution started from 1:100). The neutralizing breadth was indicated by the number of HCVpps neutralized>50%; positive neutralization was categorized as a reduction in infectivity by 50% in this HCVpp system. Spearman *R* and *P* values were indicated.
